# Cryptococcus neoformans responds to presence of Mycobacterium by diversifying its morphologies and remodelling its capsular material

**DOI:** 10.1099/jmm.0.002128

**Published:** 2026-02-23

**Authors:** Orlando Ross, Andrew Akampurira, Liliane Mukaremera, Ivy M. Dambuza

**Affiliations:** 1 Medical Research Council Centre for Medical Mycology, University of Exeter, Exeter, UK; 2 Department of Microbiology, College of Health Science, Makerere University, Kampala, Uganda

**Keywords:** capsule, co-infection, *Cryptococcus*, morphogenesis, *Mycobacterium*, virulence

## Abstract

**Introduction***. Cryptococcus neoformans* and *Mycobacterium tuberculosis* (MTb) are opportunistic pathogens that share overlapping geographical distributions and physiological niches within the human body. Both are recognized by the World Health Organization as high-priority pathogens.

**Gap Statement**. Although clinical reports of co-infections with cryptococcosis and tuberculosis are increasing, experimental studies exploring their interactions remain scarce.

**Aim**. We aimed to observationally evaluate whether *C. neoformans* isolates would alter morphology when co-cultured with *Mycobacteria* spp. and observe how these changes might alter the host immune response to *C. neoformans* cells.

**Methodology**. We cultured *C. neoformans* reference strain and clinical isolates in physiologically relevant growth media, in the presence or absence of *Mycobacterium* spp. Then, we generated alveolar-like macrophages and created a stimulation environment similar to a tuberculosis environment to perform phagocytic killing assays of *C. neoformans* cells.

**Results**. Here, we demonstrate that *C. neoformans* can grow in the presence of either heat-killed MTb antigen or the live vaccine strain, *Mycobacterium bovis* BCG. In response to the presence of mycobacteria, *C. neoformans* increased in number and exhibited enhanced virulence-associated traits, including titan cell formation, capsule enlargement and increased survival from phagocytosis.

**Conclusion**. This work provides proof of principle for a dynamic, inter-pathogen interaction that may contribute to the exacerbation of disease outcomes in settings of a co-infection.

## Introduction

*Cryptococcus neoformans* colonizes the pulmonary region through inhalation of spores and desiccated yeast cells, often found in bird guano and hollows of various tree species, including *Eucalyptus* spp., in the environment [1,3]. Pulmonary cryptococcosis results in the formation of nodules or masses in the lung, known as granulomas. In parallel, the pathogenesis of tuberculosis has a closely related cycle; aerosolized *Mycobacterium tuberculosis* (MTb) droplets are inhaled, reaching the lungs, whereby colonization and granuloma formation also occur [4,5]. Cryptococcal granulomas are often indistinguishable from those formed by MTb, and impairment of the immune system, such as in Human Immunodeficiency Virus/Acquired Immune Deficiency Syndrome (HIV/AIDS), can lead to failure to restrain growth of these pathogens within the lung, resulting in spread to other organs, including the central nervous system (CNS). Both *C. neoformans* and MTb CNS infection can result in life-threatening meningoencephalitis if not promptly treated [6,7]. While mechanisms of dissemination to extrapulmonary regions remain elusive, in both cryptococcosis and tuberculosis, it is thought that the pathogens seed distal tissues through trafficking by infected macrophages or through systemic infection when granulomas fail to contain pathogen growth [6,8,13].

The World Health Organization (WHO) classified *C. neoformans* as a ‘critical priority’ in the first-ever ‘Fungal Pathogens Priority List’ in October 2022 [14]. *C. neoformans* is estimated to be implicated in 194,000 cases of HIV-associated cryptococcal meningitis globally, with 118,000 *C*. *neoformans*-mediated meningitis (CM) related deaths [15,16]. A disease-linked mortality is estimated to be as high as 70% in low-income countries, compared to 20–30% in high-income countries [15,16]. Early-stage diagnosis of cryptococcosis is crucial for reducing mortality, but detection through rapid testing, or affordable methods that do not use PCR/RNA sequencing, is usually only employed when the disease is presenting, a sign of advanced CM and often too late [17]. Similarly, the mortality rates caused by MTb are equally devastating. For instance, MTb was the leading infectious disease single-agent cause of death globally, before the COVID-19 pandemic, with 50% mortality observed in untreated patients [18,19]. MTb is estimated to latently infect nearly one quarter of the global population, up to 1.7 billion people [18,20]. The 2022 WHO global tuberculosis report estimated that 10.6 million people became ill and 1.6 million people died from tuberculosis in 2021 alone, with 11.7% of deaths occurring in HIV-positive patients [18]. Multidrug-resistant tuberculosis incidence has also risen, further driving morbidity and mortality, alongside healthcare-associated costs [21].

Yet, mounting evidence demonstrates the increasing occurrence of *Mycobacterium* and *Cryptococcus* spp., co-infections, which is of great public-health concern, especially in sub-Saharan Africa and Asia in both HIV-positive and HIV-negative populations [22,26]. *Mycobacterium* and *Cryptococcus* spp., co-infections significantly increase the risk of death compared to *Cryptococcus* deaths alone [22]. Despite this, we have very little understanding of whether these pathogens interact with one another or whether pathogenesis or virulence is increased or hindered in a co-infection setting. We hypothesized that *C. neoformans* would be influenced by the presence of *Mycobacterium* spp., potentially upregulating virulence factors such as cell body size and capsule, as we have previously shown that bacterial cell wall components drive *C. neoformans* cell enlargement [27]. Other fungal–bacterial co-infection settings result in drastic prognostic changes and confer worse clinical outcomes, such as *Candida albicans–Staphylococcus aureus* co-infections, which have devastating consequences for the host [28]. In this study, we demonstrate that *C. neoformans* senses mycobacteria and shifts towards a more virulent state, increasing proliferation and inducing capsule and titan formation. This establishes proof-of-principle that inter-pathogen interactions may potentiate *C. neoformans* pathogenicity during co-infection settings.

## Methods

### Strains and culture conditions

*C. neoformans* and *Mycobacterium* spp. strains used in this study are summarized in Fig. S1, available in the online Supplementary Material. *C. neoformans* clinical isolates were kindly provided by Professor Kirsten Nielsen (Virginia Tech University, USA). Yeast cells were routinely grown on yeast extract peptone adenine dextrose (YPD) agar plates, following Chun and Madhani’s recipe [29], and stored at 4 °C. For routine culture, cells were incubated at 30 °C for 12–16 h overnight in 50 ml Falcon tubes with 5 ml YPD broth, recipe also by Chun and Madhani [29], at 30 °C, 150 r.p.m. Vi-cell Blu viability analyser was used to adjust for 1×10^6^ cells in 5 ml PBS solution (10×, pH 7.4, Gibco^™^, Fisher Scientific, Loughborough, UK) for the subsequent investigations. *Mycobacterium bovis* BCG (Bacillus Calmette-Guérin) was kindly provided for this study by Romey Shoesmith (MRC CMM, Exeter, UK). Glycerol stocks were inoculated onto Middlebrook 7H10 agar (M0303, Sigma-Aldrich, Dorset, UK), supplemented with 10% Tween-80 (655207, Sigma-Aldrich, Dorset, UK), at 37 °C for 21 days. These plates were then stored at 4 °C. Liquid culture was also prepared in Middlebrook 7H9 media (M0178, Sigma-Aldrich, Dorset, UK), supplemented with 10% Tween-80; 250 ml of media was added to a sterile glass conical flask aseptically. This flask was inoculated with one sterile loop of glycerol-suspended *M. bovis* stock, incubated at 30 °C and 70 r.p.m., with the media replenished weekly. A sterile conical flask was incubated with 250 ml of the same 7H9 media preparation and incubated alongside the liquid culture to demonstrate sterility of the preparation. For *M. bovis*, OD_600_ was normalized for 1×10^6^ cells in 5 ml PBS for the subsequent investigations.

### Assessing *C. neoformans* morphological changes in the presence/absence of *M. bovis* or MTb

Briefly, 50 µl of each *C. neoformans* strain inoculum was added to 12-well flat-bottomed plates, with 5 µl of *M. bovis* inoculum (seeding density of 1,000 cells per well) or 2.5 ng H37Ra heat-killed MTb (HK-MTb) (50 ng ml^−1^) added either at the initial inoculation or 24 h after *C. neoformans*, with 1 ml of human plasma-like medium (HPLM)+10% foetal bovine serum (FBS) growth media, in duplicate. Plates were incubated at 37 °C in 5% CO_2_ for 48 h, before being pelleted, washed and fixed in 4% formaldehyde in PBS for 1 h. Cells were then spun, washed and resuspended in 50 µl PBS for imaging and further analysis.

To assess the impact on cell proliferation in the presence/absence of MTb, yeast cell quantification was performed on a Vi-cell Blu viability analyser.

### India ink staining and image acquisition

India ink staining was used to observe both *C. neoformans* cell body and capsule under the microscope. Briefly, 5 µl of cell suspension was mixed with 5 µl of India Ink (Remel BactiDrop^™^, KS, USA) counterstain and observed under an Olympus CKX53 microscope and imaged with an Olympus EP50 camera using the Olympus EPview software (version 1.4 for Windows). These images were analysed in ImageJ2 (version 2.14.0 for macOS).

### Measurement of *C. neoformans* cell body and capsule sizes

As described above, India ink-stained cells were used to analyse *C. neoformans* cell body and capsule sizes. Cell body diameter and capsule were measured using Fiji, with two frames per sample and condition analysed per experimental repeat (two areas per slide/four areas analysed in total). Cells were randomly selected, with *n*≥200 cells total and *n*≥100 cells total, per H99 condition and per clinical isolate, respectively. Capsule size was calculated by taking the cell body size from the total cell diameter measured.

### Generation of alveolar-like macrophages from murine bone marrow

Mouse bone marrow cells were isolated from wild-type C57BL/6 mice (purchased from Charles River Laboratories, UK), with erythrocytes removed by RBC lysis buffer (11814389001, Sigma-Aldrich, Dorset, UK). The remaining cells were filtered through a 70 µm cell strainer. In total, 5×10^5^ bone marrow cells were seeded into each well of a 12-well plate and supplemented with Dulbecco's Modified Eagle's Medium (DMEM) (4.5 g l^−1^ glucose, 12077549, Gibco^™^, Fisher Scientific, Loughborough, UK) containing 10% FBS, 100 U ml^−1^ penicillin–streptomycin (11548876, Gibco^™^, Fisher Scientific, Loughborough, UK), 20 ng ml^−1^ Granulocyte-macrophage colony-stimulating factor (GM-CSF) (415 ml-020, Bio-techne, Abingdon, UK) and 2 ng ml^−1^ Transforming growth factor-beta (TGF-*β*) (7666 MB-005, Bio-techne, Abingdon, UK) (hereby referred to as GT media). These cells were maintained at 37 °C, 5% CO_2_ for 7 days, with no media changes. On day 7, the media was refreshed with the addition of 0.1 µM PPAR-*γ* agonist rosiglitazone (5325/10, Bio-techne, Abingdon, UK) (hereby referred to as GTR media) and incubated for a further 4 days. On day 11, non-adherent cells were washed off and discarded with Hank’s balanced salt solution (HBSS) (15266355, Gibco^™^, Fisher Scientific, Loughborough, UK) and re-supplemented with GTR media. On day 12, cells were detached by Accutase (A1110501, Gibco^™^, Fisher Scientific, Loughborough, UK) and cell scrapers and harvested as alveolar macrophage (AM)-like cells. In total, 7.5×10^7^ AM-like cells were harvested, as quantified by Vi-cell Blu viability analyser.

### Confirmation of AM-like phenotype by flow cytometry

AM-like cells were harvested as described above, fixed with 1% formaldehyde for 30 min and then incubated at 4 °C for 10 min with eFluor780 viability dye (eBioscience^™^, Fisher Scientific, Loughborough, UK). Then, cells were blocked by Fc-blocking buffer (anti-CD16/32, BD Biosciences, Wokingham, UK) for 10 min at 4 °C. Cells were subsequently stained with anti-CD11b (BUV395, BD Biosciences), anti-CD11c (BV711, BD Biosciences), anti-Siglec-F (CD170 Super Bright 436, Fisher Scientific), anti-CD45 (PerCP-Cy 5.5, BD Biosciences) and anti-F4/80 (565410, BD Biosciences) at 4 °C for 1 h. Cells were then washed with flow cytometry cell buffer containing 0.5% BSA, 5 mM EDTA with 7-AAD, before being measured by Cytek Aurora Flow Cytometer (Cytek Biosciences, Amsterdam, The Netherlands) and analysed on FlowJo (version 10.9.0 for macOS).

### Assessing the immune response to *C. neoformans* in the presence and absence of MTb by alveolar-like macrophage cells

Harvested day-11 AM-like cells were seeded into four 12-well polystyrene plates at a density of 5×10^5^ cells per well and maintained in 1 ml GTR media. Two plates were primed with 50 ng H37Ra HK-MTb (50 µg ml^−1^) and 1 ng rIFN-*γ* (485-MI, Bio-techne, Abingdon, UK) for 4 h, at 37 °C, 5% CO_2_. At 4 h, all plates were inoculated with 100 µl 2×10^6^ cells ml^−1^ H99 cells, grown in HPLM for 30 days prior to infection. These cells were filtered through 40 µm cell strainers to remove any large *C. neoformans* cells that AM-like cells would be unable to internalize, and plates were incubated for 12 h at 37 °C, 5% CO_2_. To quantify internalization of *C. neoformans*, plates were washed with HBSS to remove any suspended *C. neoformans* cells. Alveolar-like macrophages were lysed with purified water, and the resulting suspension was either plated onto YPD agar plates in serial dilutions and incubated at 37 °C for 48 h, or yeast cells were quantified using Vi-cell Blu viability analyser.

### Statistical analyses

These data were statistically analysed in GraphPad Prism 10 (version 10.0.0 for macOS). For comparisons between conditions, ANOVA analyses were carried out, alongside t-tests and Tukey’s multiple comparison tests. Statistical significance was calculated with the following classifications: * *P*≤0.05, ** *P*≤0.01, *** *P*≤0.001, **** *P*≤0.0001.

## Results

### *C. neoformans* cell body, capsule size and cell density are significantly increased by co-culture with *Mycobacterium* species

**Fig. 1.**
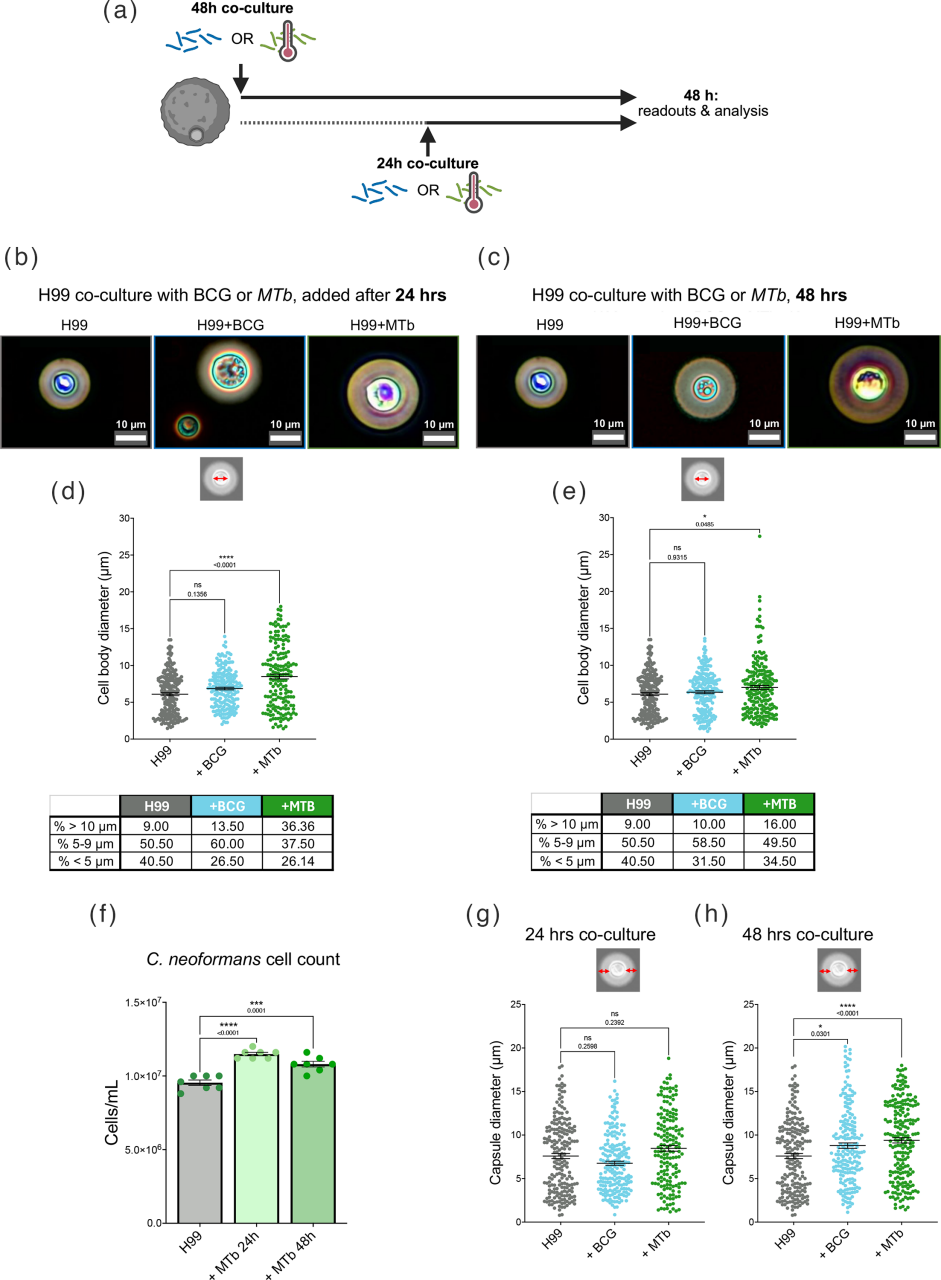
*C. neoformans* morphologies are significantly altered in the presence of *Mycobacterium* spp. (**a**) Schematic of culture conditions. *C. neoformans* H99 overnight cells incubated in HPLM supplemented with 10% FBS at 37 °C, 5% CO_2_; either alone (1×10^4^ cells per well) or with *M. bovis* (1×10^3^ cells) or MTb (2.5 ng heat-killed desiccated H37Ra) for (b) 24 h or (c) 48 h co-culture. H99 cells were counterstained with India ink, imaged on Olympus EP50 camera and mounted to Olympus CKX53 microscope. (b) and (c) show representative images of cell body diameter and percentages of different subpopulations (>10 µm, between 5 and 9 µm and <5 µm) at (d) 24 h and (**e**) 48 h. (**f**) shows cell density after co-incubation with MTb, quantified using Vi-cell Blu viability analyser. (g) (24 h) and (h) (48 h) show capsule size (diameter). Data presented are mean±sem from eight technical repeats across two biological replicates. All cell measurements were made using Fiji, graphed on GraphPad Prism, *n*=200 cells (eight technical repeats across two biological repeats). Tukey’s multiple comparisons test was used to assess statistical significance of populations as a whole; * denotes *P*≤0.05, *** denotes *P*≤0.001, **** denotes *P*≤0.0001, ‘ns’ denotes not statistically significant.

We have shown recently that *C. neoformans* produces *in vivo* morphologies when grown *in vitro* using HLPM, 5% CO_2_ and 37 °C [30]. These culture conditions provided ideal *in vitro* settings to test whether *C. neoformans* morphological switching, which is a characteristic virulence factor [31,33], was impacted by presence of mycobacteria. To assess this, we co-cultured H99 with live *M. bovis* BCG (Fig. 1a). When we assessed the overall mean cell body size within the cultures, in the presence of BCG at either 24 (Fig. 1b, d) or 48 h (Fig. 1c, e), we found no significant change compared with H99 monoculture. However, analysis of size distributions at 24 h revealed a shift in population structure: the proportions of titan cells (≥10 µm) and yeast-sized cells (5–9 µm) increased by ~4.5 and 10%, respectively, while the fraction of smaller cells (≤5 µm) decreased by ~14% (Fig. 1d). This pattern suggests that H99 senses the presence of live BCG and responds by diversifying its population heterogeneity, generating a higher proportion of yeast-sized and titan morphotypes while reducing small-cell abundance. This response was still detectable after 48 h of co-culture, although more modestly, with titan and yeast-sized cells increasing by ~1 and 8%, respectively, and the proportion of smaller cells decreasing by ~9% (Fig. 1e). These findings indicate that the interaction between *C. neoformans* H99 and live BCG promotes sustained remodelling of the fungal population structure, consistent with an adaptive sensing mechanism rather than passive morphological drift. We also examined relative to the avirulent BCG, how H99 responds to a virulent mycobacterium strain, MTb. The absence of a biosafety level 3 facility prevented the use of live MTb; therefore, HK-MTb was used, which retains complex immunogenic cell wall lipids and glycolipids associated with its virulence. Notably, co-culture of H99 with HK-MTb resulted in a statistically significant increase in the mean cell body size of the overall H99 population at both 24 (Fig. 1b, d) and 48 h (Fig. 1c, e). Analysis of population size distributions revealed a striking shift at 24 h, with the proportion of titan cells (≥10 µm) increasing by 27.4%, accompanied by a 13% reduction in yeast-sized cells (5–9 µm) and a 14% decrease in smaller cells (≤5 µm) (Fig. 1d). This pattern was still detectable at 48 h, although to a lesser extent, with titan cells increasing by 7% and yeast-sized and smaller cells decreasing by 1 and 6%, respectively (Fig. 1e). These findings indicate that exposure to virulent MTb or the increased MTb-associated molecules exposed due to heat killing resulted in pronounced and sustained restructuring of H99 population morphology, strongly favouring titan cell formation. Importantly, when we assessed the total cell counts, we observed a significant increase in H99 cell density during HK-MTb co-culture (Fig. 1f), suggesting that this morphological shift occurs in parallel with overall population expansion rather than growth suppression.

The polysaccharide capsule is a well-studied virulence determinant of *C. neoformans*, functioning both as a physical barrier and as a dynamic regulator of host–pathogen interactions [31]. Titan cells are characterized by dramatic capsule thickening associated with enhanced immune evasion and persistence in tissues [32,34]. In our previous work, we established that HLPM, 5% CO_2_ and 37 °C *in vitro* conditions reliably induced large capsule formation, closely mirroring capsule expansion observed *in vivo*, and importantly, this phenotype extended beyond titan cells to include smaller cell populations [30]. Given that both cell body enlargement and capsule remodelling contribute to pathogenic fitness, we next examined whether exposure to live BCG or HK-MTb alters capsule size in H99 populations. Capsule measurements at 24 h revealed no significant difference in capsule diameter when H99 was co-cultured with either live BCG or HK-MTb compared with monoculture (Fig. 1g). However, by 48 h, both live BCG and HK-MTb induced a significant increase in capsule thickness (Fig. 1h). This indicates that prolonged exposure to mycobacterial cues promotes capsule remodelling. Together, these data show that *C. neoformans* H99 expands its capsule in response to either attenuated or virulent mycobacterial components, suggesting that capsule remodelling is a generalized response to mycobacterial sensing rather than a virulence-specific phenomenon.

### Clinical isolates of *C. neoformans* remodel the cell body population and capsule production in the presence of live *M. bovis*

**Fig. 2.**
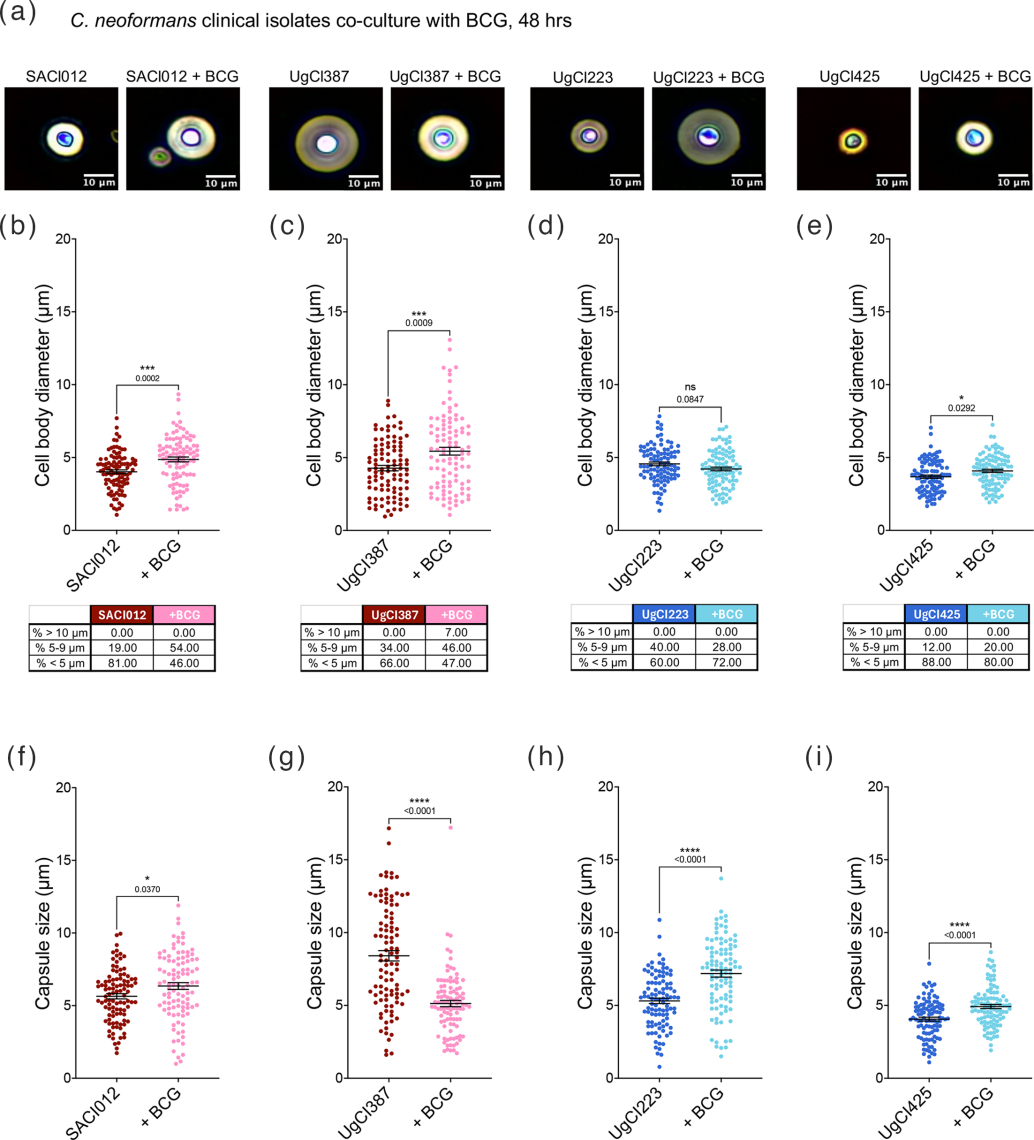
Clinical isolates of *C. neoformans* remodel the cell body population and capsule production in the presence of live *M. bovis*. High (in red) and low (in blue) virulence *C. neoformans* clinical isolates were grown overnight in YPD at 30 °C with shaking, washed with PBS and then resuspended in HPLM supplemented with FBS in 12-well plates (1×10^4^ cells per well). These cultures were then incubated with or without *M. bovis* (BCG) (1×10^3^ cells per well) in HPLM supplemented with 10% FBS at 37 °C, 5% CO_2_ for 48 h at 37 °C and 5% CO_2_. Cells were counterstained with India ink and observed on Olympus CKX53 microscope, with Olympus EP50 camera. (**a**) shows representative images of India ink-stained cells. (b)–(e) show overall percentage cell body size, and the table below each graph indicates the percentages of different *C. neoformans* subpopulation (>10 µm, between 5 and 9 µm and <5 µm). (f)–(i) show capsule diameter measured using Fiji. Data presented are from two biological replicates with at least 100 cells per replicate. Median with interquartile range shown by error bars. Mann-Whitney U test was used to measure statistical significance between *C. neoformans* strains alone and in co-culture with BCG, * denotes *P*≤0.05, ** denotes *P*≤0.01, **** denotes *P*≤0.0001.

Recent work shows that clinical isolates of *C. neoformans* modulate different disease outcomes in patients [35,37]. H99 originates from a Hodgkin’s lymphoma patient, existing in the VNI *C. neoformans* clade [38] and is not closely related to the majority of strains that are associated with HIV – the key group of patients at risk from *C. neoformans* – MTb co-infections [35,39]. To determine whether the morphological response to mycobacteria was conserved across diverse *C. neoformans* backgrounds, we examined two high-virulence clinical isolates (SACl012 and UgCl387) and two low-virulence isolates (UgCl223 and UgCl425) [35] (Table S1, Fig. S1). When co-cultured with live BCG under HLPM conditions, both SACl012 (Fig. 2a, b) and UgCl387 (Fig. 2a, c) exhibited clear increases in mean cell body size and restructuring of population architecture compared with monoculture. SACl012, which does not generate titan cells under these culture conditions, responded by markedly enriching its yeast-sized (5 and 9 µm) population, showing a ~35% increase in yeasts and a 35% reduction in smaller cells (≤5 µm) (Fig. 2b). UgCl387, by contrast, not only expanded its yeast-sized population by ~12% but also generated titan cells *de novo*, with a ~7% increase in cells ≥10 µm and a concurrent ~19% decrease in smaller forms (Fig. 2c). These findings parallel the remodelling observed in H99 and indicate that high-virulence isolates readily diversify their morphological repertoire in response to mycobacterial cues, shifting towards cell types linked to persistence and immune resistance.

The low-virulence isolates exhibited distinct responses that were not defined simply by attenuated versions of the high-virulence pattern. UgCl223 showed no significant change in mean cell size overall (Fig. 2d). However, analysis of size distributions revealed a notable increase in smaller cell forms (≤5 µm) accompanied by a decrease in yeast-sized cells, by ~12% (Fig. 2d). This redistribution is not indicative of a neutral or diminished response but instead suggests a shift towards a morphology associated with improved dissemination [40]. Small-cell morphotypes have been reported to traverse endothelial barriers more efficiently and seed distant tissues [40], implying that, in this isolate, mycobacterial sensing may promote a spread-oriented pathogenic strategy rather than immune evasion through size enlargement. UgCl425, in contrast, displayed a statistically significant increase in mean cell size (Fig. 2e), driven by an ~8% expansion of yeast-sized cells and a reduction in smaller forms, without titan cell emergence, representing a more moderate remodelling profile. Taken together, these findings demonstrate that *C. neoformans* does not adopt a uniform response to mycobacterial co-culture. Instead, each isolate shifts along a morphological trajectory that aligns with its underlying virulence programme. High-virulence isolates preferentially enriched titan and yeast morphotypes associated with immune evasion and tissue persistence, whereas low-virulence isolates either enhanced small-cell production to favour dissemination or expanded yeast-sized populations without generating titan forms. Thus, exposure to mycobacteria does not impose a single pathogenic state but rather acts as an environmental signal that amplifies strain-specific virulence strategies already embedded within the genetic identity of each isolate.

We next assessed whether clinical *C. neoformans* isolates remodel their capsule when co-cultured with mycobacteria. All isolates were grown in HPLM and co-cultured with live BCG for 48 h under host-like conditions. The high-virulence isolate SACl012 exhibited a significant increase in capsule thickness in response to BCG (Fig. 2f), mirroring the capsule expansion observed in H99. In contrast, the second high-virulence isolate UgCl387 demonstrated a significantly reduced capsule when exposed to BCG (Fig. 2g), indicating that even among isolates with similar clinical severity profiles, capsule remodelling can occur in opposing directions. For the low-virulence isolates, both UgCl223 and UgCl425 showed significant capsule enlargement following BCG co-culture compared to monoculture (Fig. 2h, i). Notably, these responses occurred despite distinct effects of BCG on cell body size and population structure, suggesting that capsule remodelling is a robust and conserved response to mycobacterial cues, whereas the direction and morphological context of that remodelling differ between isolates. Together, these findings demonstrate that exposure to mycobacteria triggers capsule restructuring across genetically and clinically diverse *C. neoformans* isolates. Rather than a uniform programme, *C. neoformans* appears to deploy isolate-specific remodelling strategies, with some isolates increasing capsule thickness and others reducing it. Because capsule architecture is a key determinant of immune evasion, dissemination and persistence, such plasticity has important implications for co-infection.

### Pre-activation of alveolar-like macrophages with IFN-*γ* and MTb cues reduces their capacity to restrict *C. neoformans* growth

**Fig. 3.**
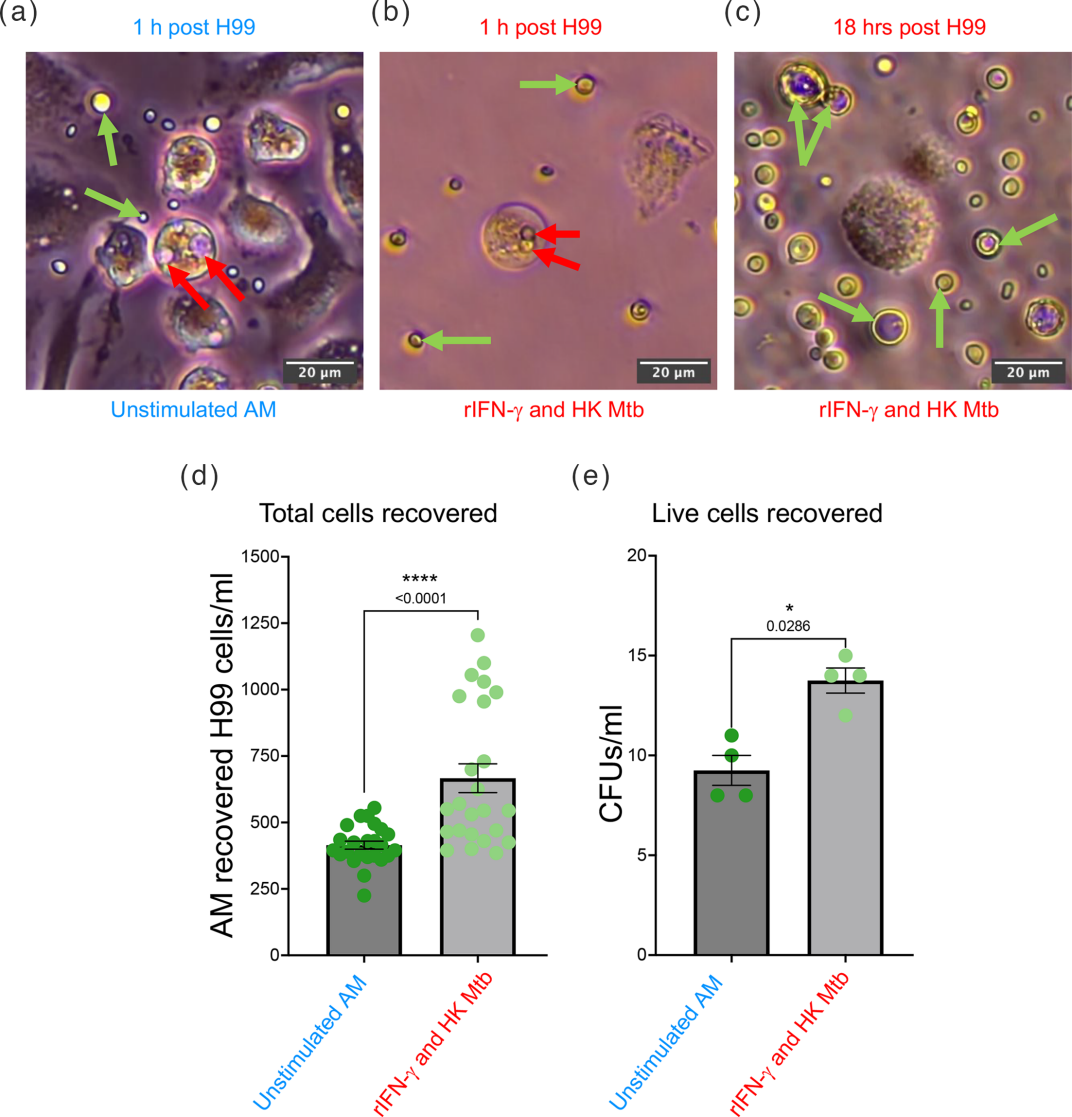
Pre-activation of alveolar-like macrophages with IFN-*γ* and MTb cues reduces their capacity to restrict *C. neoformans* growth. Bone marrow cells from C57BL/6 mice were used to generate alveolar-like macrophages. AMs were then stimulated with HK-MTb H37Ra and recombinant IFN-*γ* for 4 h at 37 °C, 5% CO_2_. Briefly, 5×10^5^ unstimulated and stimulated AMs/well were challenged with 2×10^5^ *C. neoformans* H99 cells at an m.o.i. of 2:5 *C*. *neoformans* cells:AMs. AMs were imaged on an Olympus CKX53 microscope with EP50 camera, at 20× magnification with 100×100 µm panels used to show: (**a**) unstimulated AM response to 1 h H99 challenge, (**b**) rIFN-*γ* and HK MTb-stimulated AM response to H99 and (**c**) stimulated AM response 18 h post-H99 challenge. Red arrows indicate internalized H99 cells; green arrows indicate external H99 cells in suspension. At 18 h post-infection, supernatant was removed and AMs were washed with HBSS to remove any H99 cells that were not internalized by AMs. Then, AMs were lysed with dH_2_O, and (d) total H99 cells were quantified using a Vi-cell Blu viability analyser or (e) plated onto YPD agar to count live H99 cells that were internalized by AMs. Data presented are mean±sem from 1 biological replicate with 24 technical replicates for total cells and 4 technical replicates for c.f.u.s. Mann-Whitney U test was used to compare *C. neoformans* cell number from unstimulated versus rIFN-*γ* and HK MTb-stimulated AMs; * denotes *P*≤0.05, **** denotes *P*≤0.0001.

Pulmonary alveolar macrophages are among the first immune cells to encounter *C. neoformans* in the lung. To determine whether the morphological and population-level changes induced by mycobacterial exposure alter macrophage-fungal interactions, we next assessed the ability of alveolar-like macrophages (AMs) to restrict *C. neoformans* growth. AMs were generated from murine bone marrow cells by cytokine differentiation [41] (Fig. S1A and B), and flow cytometric analysis confirmed acquisition of a CD11b^high^, CD11c^+^, Siglec-F^+^, F4/80^high^ phenotype characteristic of resident AMs (Fig. S1C). AMs were pre-stimulated with heat-killed HK-MTb and recombinant IFN-γ [42,44], to mimic a type-1 immune environment shaped by presence of MTb infection, which are known to confer anti-microbial activity to macrophages [45,46]. AM morphology was monitored throughout co-culture (Figs 3a-c and S1D). After 18 hours, significantly higher numbers of viable *C. neoformans* cells were recovered from AMs pre-stimulated with IFN-γ and HK-MTb compared with unstimulated AMs (Fig. 3d-e). These data show that AMs conditioned with mycobacterial signals are less effective at restricting *C. neoformans* growth. In the context of the morphological remodelling and increased capsule expansion in response to mycobacterial sensing; this result indicates that *C. neoformans* exposed to mycobacterial environments can persist and expand even within macrophages that display a type-1, classically activated phenotype.

## Discussion

Cryptococcosis and tuberculosis co-infections are increasingly recognized in clinical settings, particularly among individuals with compromised immunity, yet remain poorly understood and frequently underdiagnosed due to overlapping pulmonary and neurological presentations [47,50]. While the majority of available evidence derives from retrospective and post-mortem case reports, our study provides direct experimental evidence that *C. neoformans* can sense and respond to mycobacterial signals, undergoing morphological and functional changes consistent with enhanced virulence potential.

By culturing *C. neoformans* under physiologically relevant conditions [30], we observed that exposure to either live BCG or HK-MTb results in population-level remodelling of fungal morphology, including increased cell body size and, in the case of virulent mycobacterial stimuli, robust induction of titan cell formation. Titan cells are a well-established virulence phenotype characterized by increased stress resistance, impaired phagocytic clearance and altered immune activation [27,32, 33]. That this morphotype was induced even in the absence of live mycobacterial replication suggests that *C. neoformans* is responding to conserved mycobacterial-associated molecular patterns, rather than host-derived cues alone. Notably, bacterial cell wall motifs have been shown to trigger the formation of titan cells previously [27], supporting a model in which inter-kingdom microbial cues dynamically modulate fungal virulence traits. The observed differences in titanization between high-virulence strains SACI012 and UgCI387 could be due to differences in their virulence; previous work established that the time to 80% mortality in murine models was 18 days for SACI012 and 37 days for UgCI387, and that strain SACI012 was unable to form titan cells *in vitro* or *in vivo*, whereas UgCI387 could undergo titanization *in vivo* [35].

Capsule remodelling was similarly enhanced across clinical isolates following mycobacterial exposure, although the direction and magnitude varied by strain. This strain-dependent variation aligns with clinical observations that differences in capsule architecture underlie distinct dissemination trajectories and immune evasion strategies [31,33, 51]. Importantly, increased capsule thickness is predicted to reduce opsonophagocytic clearance [52,53], reinforce anti-inflammatory polarization [53] and, if such changes occur in the CNS, potentially exacerbate intracranial pressure [51], all of which may worsen disease severity during co-infection.

Secreted mycobacterial components, or those located on the cell wall, could be driving these observed changes. The trehalose biosynthesis pathway is crucial for stress tolerance in *C. neoformans* [54,55] and has been hypothesized to stabilize *C. neoformans* plasma membrane [56]. Trehalose dimycolate is an abundant *Mycobacterium* spp. cell wall component [57] where the generation at the mycomembrane results in the release of free trehalose [58]. *C. neoformans* cells could be taking advantage of the increasing trehalose concentrations and scavenging this molecule. The inorganic anion, phosphate, is required by both *C. neoformans* and MTb for cellular structure and survival [59,61]. If both of these species are competing for available phosphate in a system, it is likely that *C. neoformans* capsule remodelling and virulence expression could increase [59]. As such, this model would benefit from metabolomics and transcriptomics to understand if the observations are due to secreted metabolites or contact-mediated changes to *C. neoformans*.

The functional consequence of these morphological changes was further supported by macrophage interaction assays. When alveolar-like macrophages were primed with IFN-*γ* and MTb components, to mimic a lung environment shaped by mycobacterial infection [42,43], they internalized greater numbers of *C. neoformans* without enhanced killing. Given that *C. neoformans* can survive and replicate intracellularly, particularly within alternatively activated macrophages [11,52], this suggests that a tuberculosis-influenced immune environment may inadvertently facilitate fungal persistence. We chose to perform this assay with a low m.o.i. of 2:5 *C*. *neoformans* cells:alveolar-like macrophages so that the majority of *C. neoformans* cells would be internalized and, if any intracellular replication did occur, the wells would not experience *C. neoformans* outgrowth.

In this context, *C. neoformans* does not merely tolerate macrophage uptake but may use the intracellular niche as a protected proliferative compartment, a phenomenon consistent with previous work showing intracellular expansion and non-lytic exocytosis [11,12, 52]. Dectin-1, the canonical C-type lectin receptor responsible for recognition of pathogenic fungi within the host, playing a key role in antifungal immunity, was recently described as being indispensable for susceptibility to mycobacterial infections by recognizing *α*-Glucan in the mycobacterial capsule [62]. Interestingly, the polysaccharide *C. neoformans* capsule limits the recognition of the fungus by Dectin-1 signalling [63]. By reducing the capsule in strain UgCI387 (Fig. 2g), co-infection could result in increased recognition and host response to the fungus and could therefore confer increased survival from cryptococcosis to the host. Additionally, the C-type lectin receptor Dectin-2 is expressed on myeloid immune cells, with Dectin-2 ligands described recently, including MP98 in *C. neoformans* [64,65] and mannose-capped lipoarabinomannan in MTb [66], indicating that host recognition of these pathogens could, in part, be recognized by the same receptors. If these pathogens are involved in active co-infections, host control could be implicated and overwhelmed if these mechanisms rely on the same recognition receptors and downstream signalling. Translation of the findings laid out in this manuscript into *in vivo* models is therefore imperative to understand the impact of co-infection on host control of infection and immunity.

Recent work has shown that other lung-resident bacteria, such as *Pseudomonas aeruginosa*, can inhibit *Cryptococcus* spp. growth in a contact-dependent fungicidal manner [67], which also translates to *in vivo* models whereby previous exposure to *P. aeruginosa* is protective against subsequent *Cryptococcus gattii* infection [68]. As such, it is highly important to consider *C. neoformans* infections in the context of other, prevalent and devastating, diseases.

Future work should include quantitative analysis of *C. neoformans* components, such as hydrophobicity of capsule [69], or cell wall/capsule structure (by electron microscopy) and proteomics/transcriptomics [70] to understand the drivers of these morphological changes at a cellular and biochemical level. Whilst out of the scope of this study, these investigations would strengthen our findings and help to bridge a critical knowledge gap that has the potential to translate into patient treatment and improving clinically important outcomes.

Together, these findings support a model in which sensing of mycobacteria by *C. neoformans* enhances its pathogenic capacity, potentially reshaping the pulmonary immune landscape in ways that facilitate cryptococcal adaptation, persistence and dissemination. These findings could translate into the clinic, with increased titanization and enhanced capsule production linked to heightened antifungal resistance and *C. neoformans* persistence [71]. Thus, we would be interested to carry out screening on patients with active tuberculosis infection for cryptococcal infection in endemic settings. This phenomenon parallels other examples of inter-pathogen modulation of virulence, including *S. aureus*–*Ca. albicans* [28,72] and *Acinetobacter baumannii*–*Cryptococcus* interactions [73], where microbial sensing promotes capsule or biofilm remodelling to enhance persistence.

## Conclusion

While this study establishes that *C. neoformans* can sense and respond to mycobacterial cues in ways that enhance virulence-associated phenotypes, several key questions remain. The specific mycobacterial components or metabolic signals responsible for initiating these morphological transitions are not yet known; identifying the molecular pathways through which *C. neoformans* detects and interprets mycobacterial presence will be essential to determine whether this response is mediated by surface receptor signalling, secreted factors or direct cell–cell contact. Additionally, although our macrophage assays indicate that mycobacterial exposure primes *C. neoformans* for increased intracellular persistence, *in vivo* studies are required to determine whether these adaptations translate to altered dissemination dynamics or severity of disease during co-infection. Sequential and simultaneous infection models, particularly in lung-directed systems, will be valuable for defining how the order and timing of pathogen encounter shape disease outcome. Finally, given that both tuberculosis and cryptococcosis co-occur in regions with limited access to advanced diagnostics, understanding whether co-infection contributes to treatment failure, delayed clearance or relapse may have direct clinical relevance. Together, these lines of investigation will help clarify how inter-pathogen interactions shape infection trajectories and may ultimately inform therapeutic or diagnostic strategies for managing co-infection in high-burden settings.

## Supplementary material

10.1099/jmm.0.002128 Fig. S1.

## Abbreviations

AM: alveolar macrophage
BCG: Mycobacterium bovis Bacillus Calmette-Guérin
CM: *Cryptococcus neoformans*-mediated meningitis
CNS: central nervous system
DMEM: Dulbecco's Modified Eagle's Medium
FBS: foetal bovine serum
GM-CSF: granulocyte-macrophage colony-stimulating factor
HBSS: Hank’s balanced salt solution
HIV: human immunodeficiency virus
HK-MTb: heat-killed *Mycobacterium tuberculosis*
HPLM: human plasma-like medium
MTb: *Mycobacterium tuberculosis*
TGF-β: transforming growth factor-beta
WHO: World Health Organization
YPD: yeast peptone dextrose
