# Supplementary material for: Cryptococcus neoformans responds to presence of Mycobacterium by diversifying its morphologies and remodelling its capsular material
Source: J Med Microbiol. 2026 Feb 23;75(2):002128. doi: 10.1099/jmm.0.002128 (PMC12927488; doi:10.1099/jmm.0.002128)
Supplement: Fig. S1. [file jmm-75-02128-s001.pdf]

Supplementary Table 1. The table shows the list of strains used within this study, consisting of *C. neoformans* reference strain H99, alongside four clinical isolates obtained from patients in Mulago Hospital, Kampala, Uganda or GF Jooste Hospital, Cape Town, South Africa[31], one M. bovis reference strain (Institut Pasteur, Paris, France), and one heat-killed reference isolate of M. tuberculosis (ATCC 25177, Rockville, MD, USA). In the text, the strains are referred to indicate the location where they originate, e.g UgCl = Ugandan clinical isolate or SACl = South African clinical isolate.

Species/strain	Source	Virulence attributes	Country of Origin
C. neoformans			
H99 (ATCC 208821)	Laboratory reference	High	USA
012	Clinical isolate	High(30)	South Africa
223	Clinical isolate	Low(30)	Uganda
387	Clinical isolate	High(30)	Uganda
425	Clinical isolate	Low(30)	Uganda
M. bovis			
Pasteur strain	Laboratory reference	Low	France
M. tuberculosis			
H37Ra (ATCC25177)	Heat-killed reference	Attenuated	USA

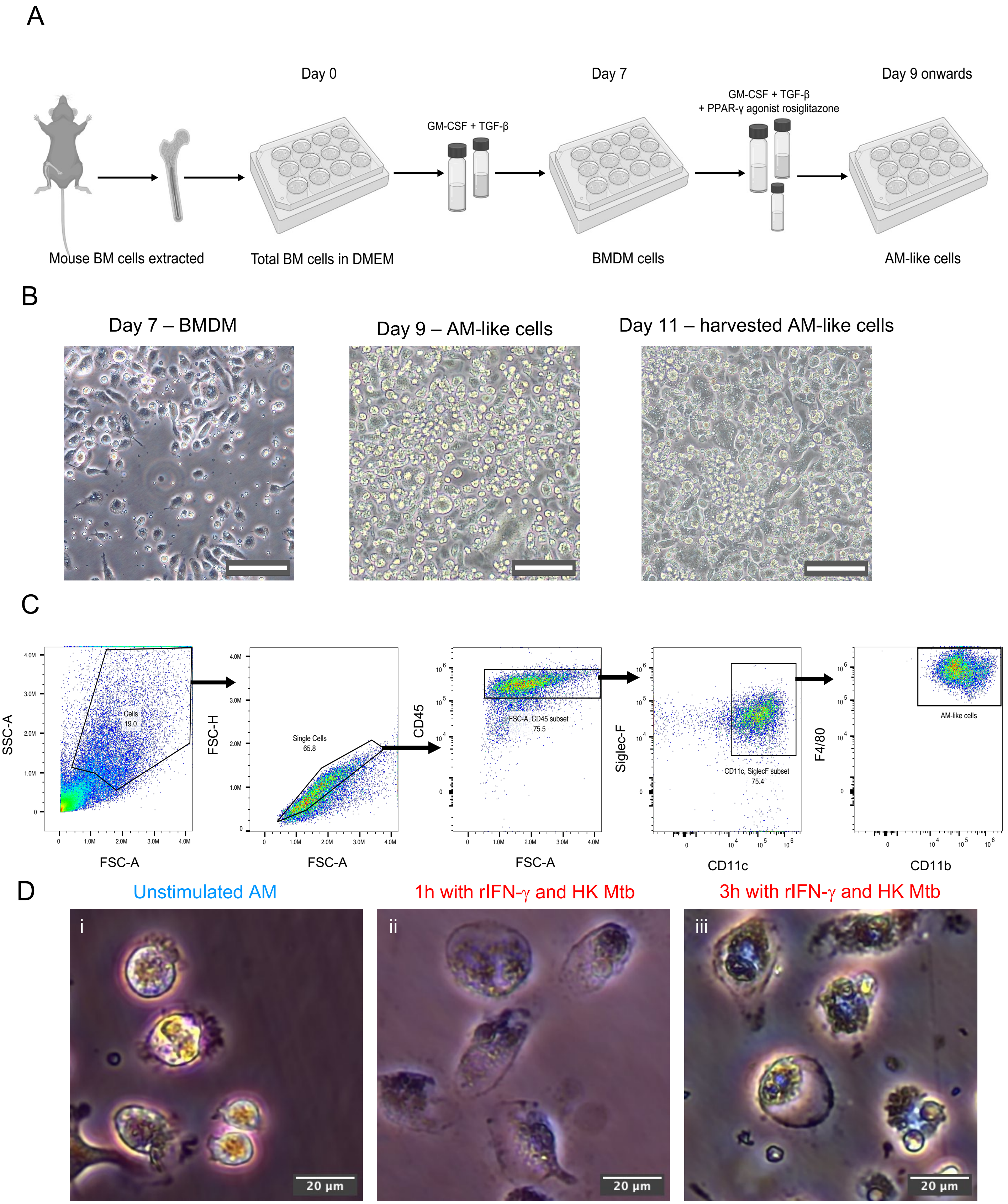

Supplementary Figure 1. The generation of alveolar-like macrophages, validation and stimulation. Bone marrow cells from C57BL/6 mice were used to generate alveolar-like macrophages (AMs). (**A**) shows the workflow (image generated using BioRender.com). (**B**) Shows morphological changes over time during AM cell differentiation, observed on Olympus CKX53 microscope, magnification = 40x, scale bar = 50 µm. (**C**) AM differentiation was confirmed by flow cytometry staining and gating for CD11c$^+$, Siglec-F$^+$, F4/80^{high}, CD11b^{high} populations. AMs were then stimulated with heat-killed M. tuberculosis H37Ra and recombinant IFN-γ for 4 hours at 37ºC, 5% CO_2. (**D**) Shows AMs morphology imaged on an Olympus CKX53 microscope with EP50 camera, at 20x magnification. The 3 images represent unstimulated AMs (left), rIFN-γ and HK Mtb-stimulated AMs after 1h stimulation (centre), and rIFN-γ and HK Mtb-stimulated AMs after 3h stimulation (right).
